# Supplementary material for: Terminating contamination: large-scale search identifies more than 2,000,000 contaminated entries in GenBank
Source: Genome Biol. 2020 May 12;21:115. doi: 10.1186/s13059-020-02023-1 (PMC7218494; doi:10.1186/s13059-020-02023-1)
Supplement: Supplementary file 3 — Additional file 3 Supplementary materials. Contains Figures S1–S3 and Listing S1. [file 13059_2020_2023_MOESM3_ESM.pdf]

Terminating contamination: large-scale search identifies more than 2,000,000 contaminated entries in GenBank

Martin Steinegger[1]**, Steven L. Salzberg** [1,]

[1]Center for Computational Biology, Johns Hopkins University, Baltimore, MD, USA

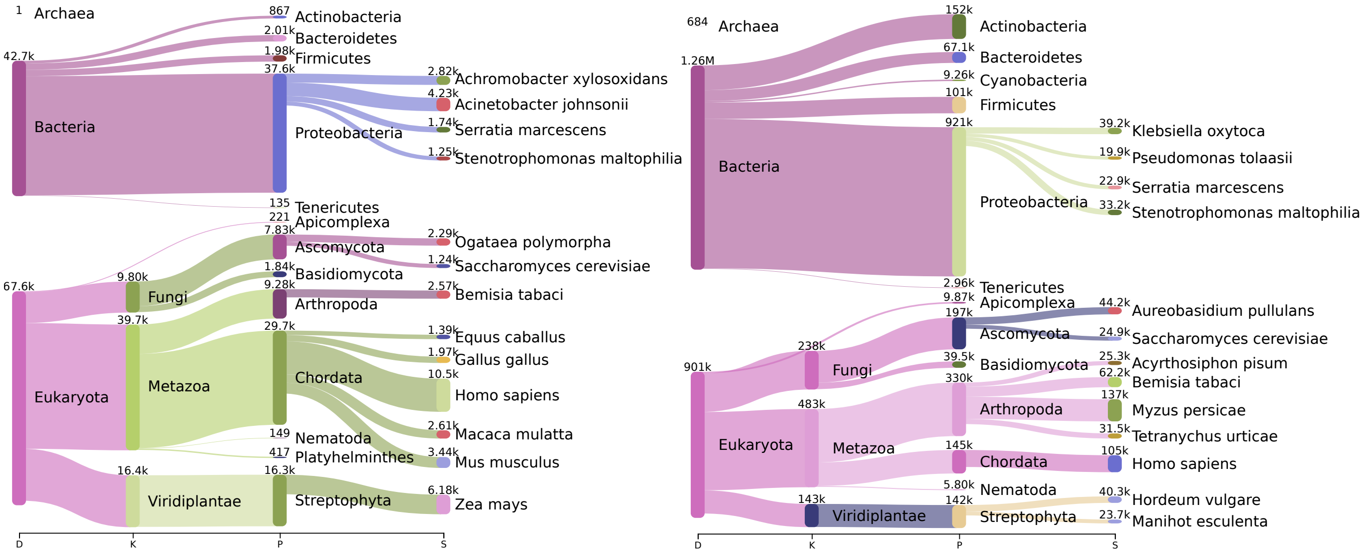

Supplementary Figure 1: **Sanksey plot of most contaminating species in RefSeq and GenBank. left** Sanksey plot five kingdoms: Bacteria&Archaea, Fungi, Metazoa, Viridiplantae and other Eukaryotes. **right** Distribution of contaminating species in GenBank.

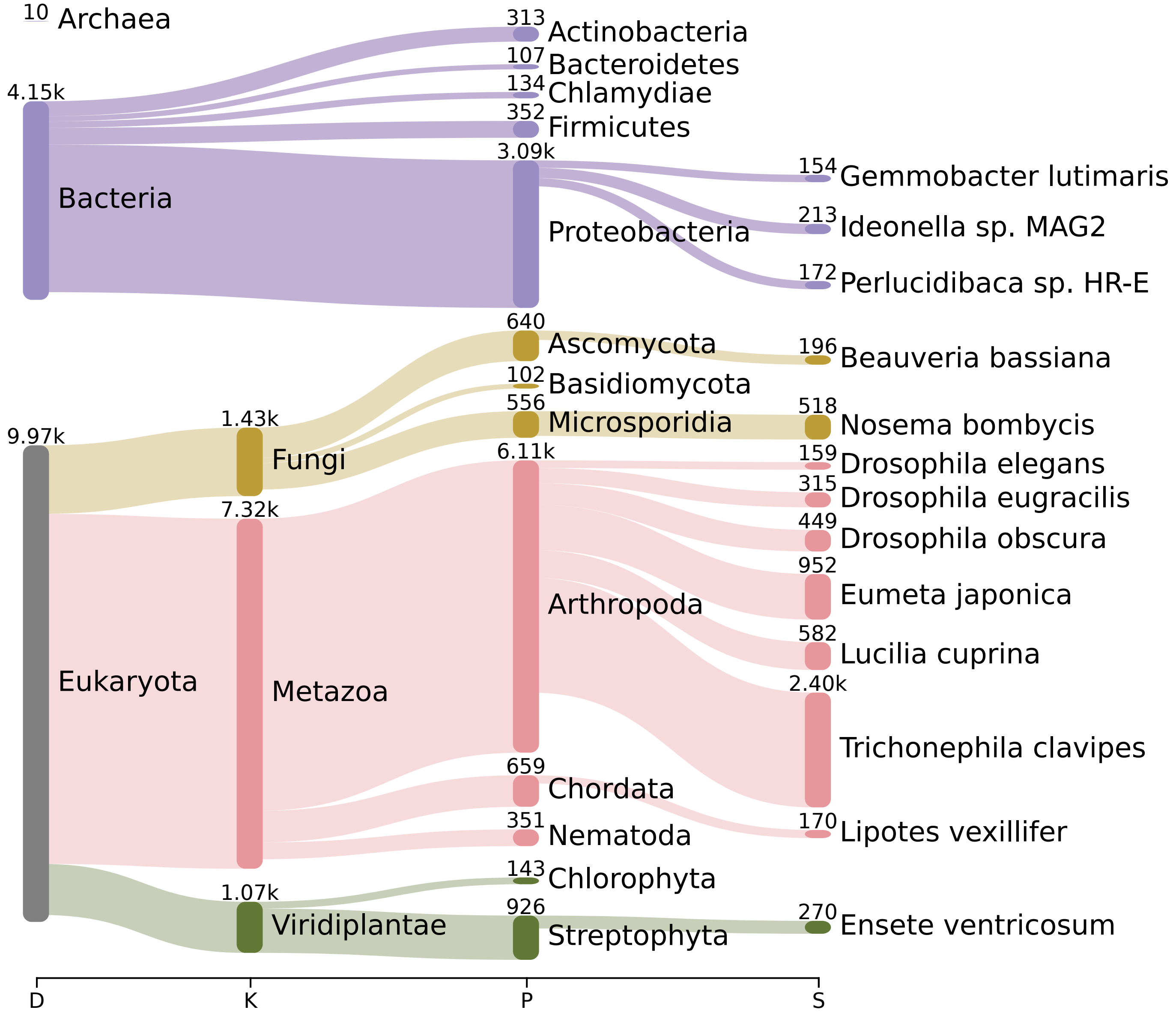

Supplementary Figure 2: Contamination in the NR database. Predicted contamination in NR protein database across five kingdoms. (Note that the contamination in the Eumeta japonica genome, visible in the figure, is being removed based on the data shown here.)

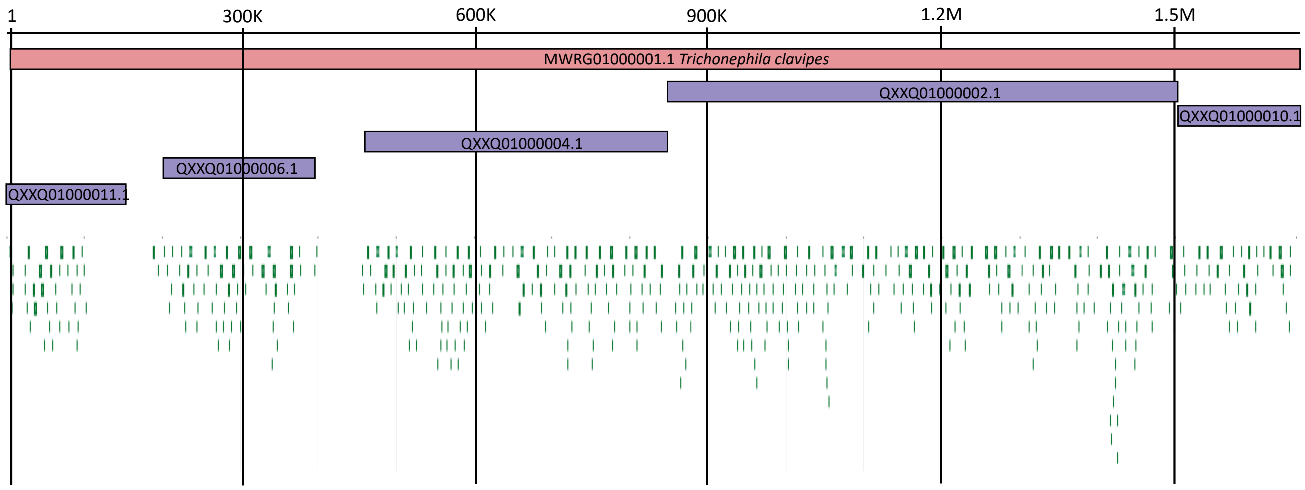

Supplementary Figure 3: **Longest contaminated contig of *Trichonephila clavipes*.** Alignment of the longest contaminated contig `MWRG01000001.1` of *T. clavipes* (red) and the genome *Gemmobacter sp. YJ-T1-11* (voilet). The green boxes are the annotations of *T. clavipes*, the contig encodes 490 proteins. The annotation are from the NCBI genome browser.

```
// (c) Script to reproduce RefSeq an NR results
// wget, BLAST and conterminator has to be installed first
// e.g. using conda
conda install -c conda-forge -c bioconda mmseqs2
conda install -c conda-forge -c bioconda blast

// How to search NR for protein contamination
wget ftp://ftp.ncbi.nlm.nih.gov/blast/db/nr.*.tar.gz
blastdbcmd -db nr -entry all > nr.faa
blastdbcmd -db nr -entry all -outfmt "%a %T" > nr.faa.taxidmapping
conterminator protein nr.fna  nr.faa.taxidmapping nr_conterm tmp

// How to search RefSeq for contamination
wget ftp://ftp.ncbi.nlm.nih.gov/blast/db/v4/refseq_genomic_v4.*.tar.gz
blastdbcmd -db refseq_genomic_v4 -entry all > refseq_genomic_v4.fna
blastdbcmd -db refseq_genomic_v4 -entry all -outfmt "%a %T" > refseq_genomic_v4.fna.taxidmapping
conterminator dna refseq_genomic_v4.fna  refseq_genomic_v4.fna.taxidmapping refseq_genomic_conterm tmp
```

Supplementary Script 1: **Script to download and run NCBI NR and RefSeq database**
