# Supplementary material for: Terminating contamination: large-scale search identifies more than 2,000,000 contaminated entries in GenBank
Source: Genome Biol. 2020 May 12;21:115. doi: 10.1186/s13059-020-02023-1 (PMC7218494; doi:10.1186/s13059-020-02023-1)
Supplement: Supplementary file 5 — Additional file 5 Review history. [file 13059_2020_2023_MOESM5_ESM.docx]

Review History

**First round of review**

**Reviewer 1**

**Were you able to assess all statistics in the manuscript, including the appropriateness of statistical tests used?**

Yes: I have assessed all statistics in the manuscript.

**Were you able to directly test the methods?**

Yes

**Comments to author:**

Steinegger and Salzberg address a very important issue of contaminations in public genome databases, by rigorously identifying plausible contaminations by very strict measures (95% identity to other published sequence) and developing a highly efficient software to execute such screening. It is a necessary task for the community to keep the public database contamination-free, and I thank the authors for undertaking this effort. Provided list of accession numbers is extremely useful to filter and update the genomes contigs, and the fact that the overall contamination rate is 0.54%~0.34% where majority of such contaminations is derived from a small number of highly contaminated genomes is somewhat reassuring.

I have only a few specific comments.

1. Contents of the Supplementary Files are not obvious. The manuscript only says "Identifiers of the contaminated sequences are available in Supplemental Files 1&2.", but these lists actually do not contain, for example, MWRG01000001 contig of Trichonephila. More information as the ones provided in the FTP server, or a more detailed legend would be helpful.

2. The files provided on the FTP server is very useful. Perhaps these files could be shared on FigShare or some other data repository?

3. pg.4 L50-55 "All contigs align to multiple positions in the domestic sheep Ovis aries genome; the fragments align to chromosome 15 (NC 040266.1) with a sequence identity greater than 94 % with nearly complete coverage." This sentence is not clear. What does "All contigs" represent? Is it the contigs shorter than 1kbp harboring one of the 185 sequences in the largest cluster? These were short contigs, but also align to the sheep genome?

4. I think the paper nicely show that the method has very low false positives, given the screening with high-quality genomes. However, how about false-negatives? Overall contamination rate of 0.54%~0.34% is presumably conservative, and some upper-bound estimate may be informative.

5. I have noticed a large contamination pointed out in Eumeta japonica genome in Fig S2, and contacted the authors since I found this paper also on biorxiv, and the author has already contacted DDBJ to remove the corresponding contigs.

**Reviewer 2**

**Were you able to assess all statistics in the manuscript, including the appropriateness of statistical tests used?**

Yes: Looks good.

**Were you able to directly test the methods?**

No

**Comments to author:**

In "Terminating contamination: large-scale search identifies more than 2,000,000 contaminated entries in GenBank", the authors discuss a new method called Conterminator that identifies cross-kingdom contamination in genome assemblies. To date, the majority of published methods used to identify genomics contamination require the user to go back to the reads from the original sequencing data, and thus are computationally intensive and intractable for large-scale analyses (e.g. searching for contamination in all of GenBank). The authors present a tool that addresses this gap, and quickly finds contamination in genomes and assemblies. This tool is needed in the community, and the analyses presented in the paper shed much needed light on contamination issues in reference databases. The tool is conda installable, and the github repo README is clear enough for a user to begin using the tool. We also find it helpful that lists of contaminants were made available by the authors.

Major concerns:

+ Although the github repo is available for the tool, we did not find an accompanying github repo for the analyses generated for this paper. We think this is very important for repeatability, and to be able to assess the methods used herein. However, we conducted the rest of this review using the text alone.

+ The authors do not note that their tool only works for cross-kingdom contamination in the abstract. This is misleading and should be corrected. Further, it would be helpful if this were added early in the introduction as well.

+ We are puzzled by combining bacteria and archaea into one "kingdom" as this is not (to the best of our knowledge) supported by any modern taxonomic or phylogenetic analyses. This also needs to be present in the introduction, we think.

+ The authors state that "systematic approaches to detect contamination are limited by computational costs of comparing every submitted genome against all other known genomes." However, the authors do not include a discussion of sketching methods, which make these comparisons feasible. SRA Taxonomy Analysis Tool in particular should be mentioned, as it has been integrated with the NCBI SRA and scales to all sequence libraries in the SRA.

+ There is a potential problem with directionality of contamination. E.g. it is sometimes difficult to know which genome contains the contaminated sequence when there are cross-kingdom matches. The authors' analyses address this question sufficiently and cleverly, and the protein sequence analysis technique addresses this question by predicting kingdoms with fewer members in the cluster as contaminated. However, the other portions of conterminator do not, and if this is not addressed by a similar method as is used by the protein sequence analysis, should be highlighted in the text as a significant shortcoming of the method.

+ There is no discussion of cross-kingdom horizontal gene transfer until the methods section, and what these events may look like to conterminator. It would be helpful to have this discussion moved forward in the paper, and to see what cross-kingdom horizontal gene transfer looks like to conterminator.

Minor concerns:

+ It would be helpful to know what types of contaminating sequences are contained in the BLAST database (currently referred to as "broader array of contaminating sequences"). Are these E. coli, PhiX, etc.?

+ The authors found Acidithiobacillus thiooxidans as a contaminant of the human reference genome. It would be interesting to see if this sequence is in any other human genomes, e.g. 1000 genomes project.

+ It is exciting that the Turkey genome is now cleaned up. Is this scalable to the rest of the contaminated genomes? If not, perhaps make a note about that?

+ how was a k-mer of length 24 selected for the selected k-mers from 1000bp windows?

+ It should be noted outside of the methods that Archaea and Bacteria are merged into one kingdom, and that environmental samples are excluded.

+ We think the authors meant the section header, "Predict contig length by find scaffolding boundaries" to read, "Predict contig length by finding scaffolding boundaries"

**Authors Response**

**Point-by-point responses to the reviewers’ comments:**

Reviewer reports:

Reviewer #1: Steinegger and Salzberg address a very important issue of contaminations in public genome databases, by rigorously identifying plausible contaminations by very strict measures (95% identity to other published sequence) and developing a highly efficient software to execute such screening. It is a necessary task for the community to keep the public database contamination-free, and I thank the authors for undertaking this effort. Provided list of accession numbers is extremely useful to filter and update the genomes contigs, and the fact that the overall contamination rate is 0.54%~0.34% where majority of such contaminations is derived from a small number of highly contaminated genomes is somewhat reassuring.

*Thank you for the supportive comment.*

I have only a few specific comments.

1. Contents of the Supplementary Files are not obvious. The manuscript only says "Identifiers of the contaminated sequences are available in Supplemental Files 1&2.", but these lists actually do not contain, for example, MWRG01000001 contig of Trichonephila. More information as the ones provided in the FTP server, or a more detailed legend would be helpful.

*We have added the contaminated protein identifier to the FTP (ftp://ftp.ccb.jhu.edu/pub/data/conterminator/nr.ids.gz and ftp://ftp.ccb.jhu.edu/pub/data/conterminator/nr.gz) and FigShare. We also added the identifier to Supplemental File 3.*

*The contig MWRG01000001 is not contained in the RefSeq results since it is longer than 20kb. We mentioned it in the manuscript because it contains the highest count of contaminated proteins.*

2. The files provided on the FTP server is very useful. Perhaps these files could be shared on FigShare or some other data repository?

*Thank you for the recommendation. We uploaded it to FigShare and add the link to the “Data availability” section (https://figshare.com/projects/Conterminator/77346).*

3. pg.4 L50-55 "All contigs align to multiple positions in the domestic sheep Ovis aries genome; the fragments align to chromosome 15 (NC 040266.1) with a sequence identity greater than 94 % with nearly complete coverage." This sentence is not clear. What does "All contigs" represent? Is it the contigs shorter than 1kbp harboring one of the 185 sequences in the largest cluster? These were short contigs, but also align to the sheep genome?

*We rewrote the sentence and it reads now:*

*"All short bacterial contigs containing the 185 proteins align to multiple positions in the domestic sheep Ovis aries genome; ..."*

4. I think the paper nicely show that the method has very low false positives, given the screening with high-quality genomes. However, how about false-negatives? Overall contamination rate of 0.54%~0.34% is presumably conservative, and some upper-bound estimate may be informative.

*We were intentionally conservative in our methods. It's probably not possible to estimate the upper bound without doing some extensive additional studies, so we didn't attempt that at this time.*

5. I have noticed a large contamination pointed out in Eumeta japonica genome in Fig S2, and contacted the authors since I found this paper also on biorxiv, and the author has already contacted DDBJ to remove the corresponding contigs.

*This is good to learn. We have added a note to the caption of Figure S2 pointing out that the contamination in Eumeta japonica is being removed based on our report. The note reads: "(Note that the contamination in the Eumeta japonica genome, visible in the figure, is being removed based on the data shown here.)"*

Reviewer #2: In "Terminating contamination: large-scale search identifies more than 2,000,000 contaminated entries in GenBank", the authors discuss a new method called Conterminator that identifies cross-kingdom contamination in genome assemblies. To date, the majority of published methods used to identify genomics contamination require the user to go back to the reads from the original sequencing data, and thus are computationally intensive and intractable for large-scale analyses (e.g. searching for contamination in all of GenBank). The authors present a tool that addresses this gap, and quickly finds contamination in genomes and assemblies. This tool is needed in the community, and the analyses presented in the paper shed much needed light on contamination issues in reference databases. The tool is conda installable, and the github repo README is clear enough for a user to begin using the tool. We also find it helpful that lists of contaminants were made available by the authors.

*Thank you for your encouraging comment.*

Major concerns:

+ Although the github repo is available for the tool, we did not find an accompanying github repo for the analyses generated for this paper. We think this is very important for repeatability, and to be able to assess the methods used herein. However, we conducted the rest of this review using the text alone.

*We agree that reproducibility is very important. However, our methods used the BLAST databases of RefSeq/NR as a key component of the experiments, and they are not version-controlled. So, we have provided a script in the Supplement (Supplementary Listing 1) that will allow others to run the same experiment with RefSeq/NR, although note that they'll get whatever the current version of RefSeq/NR is if they do that. The script will download RefSeq/NR for them.*

+ The authors do not note that their tool only works for cross-kingdom contamination in the abstract. This is misleading and should be corrected. Further, it would be helpful if this were added early in the introduction as well.

*This is not a limitation of the tool: we used it for cross-kingdom contamination in order to be very cautious (see comments from Reviewer 1 Question 4). We could easily use it to identify, e.g., cross-phylum contamination, but we chose not to do that.*

*We explain this now in the Discussion. Previously, the Discussion read*

*as follows:*

*"We used a conservative approach that only considered a sequence to be a contaminant if it had a near-identical match to a species in an entirely different kingdom from the source; e.g., a bacterial sequence found in an animal genome or vice versa."*

*We edited to read as follows:*

*"We used a conservative approach that only considered a sequence to be a contaminant if it had a near-identical match to a species in an entirely different kingdom from the source; e.g., a bacterial sequence found in an animal genome or vice versa. However, our software has a parameter (--kingdom) that allows one to identify contamination across phyla or other taxonomic levels as well."*

+ We are puzzled by combining bacteria and archaea into one "kingdom" as this is not (to the best of our knowledge) supported by any modern taxonomic or phylogenetic analyses. This also needs to be present in the introduction, we think.

*We realize that these are different kingdoms. In the original manuscript, we explained that*

*"Note that we merged Bacteria and Archaea for simplicity."*

*We have revised this to be clearer, and moved it to the Discussion which reviewer 2 request. It now reads:*

*"Note that to simplify our analysis, we merged Bacteria and Archaea, which are two distinct kingdoms, into one group. Thus, we do not detect contamination between these two kingdoms. Also note that we excluded environmental (metagenomic) samples from our analysis. We excluded viruses because some of them can integrate their genomes into other organisms, making it hard to distinguish contamination from genuine artifacts of viral integration."*

+ The authors state that "systematic approaches to detect contamination are limited by computational costs of comparing every submitted genome against all other known genomes." However, the authors do not include a discussion of sketching methods, which make these comparisons feasible. SRA Taxonomy Analysis Tool in particular should be mentioned, as it has been integrated with the NCBI SRA and scales to all sequence libraries in the SRA.

*We couldn't find a manuscript describing the SRA Taxonomy Analysis Tool, but we did find "Large-scale sequence comparisons with sourmash" (2019) by Pierce et al. on sketching to compare genomes. These methods are great for detecting a whole genome (or draft assembly, etc) that has been mis-labelled, but they are not well suited for finding a small contig in a draft assembly that is from a contaminant, which is the target of Conterminator. We added a reference to the sketching methods in the introduction on line 47, p. 2, col 1.*

*"Other fast methods such as Mash [20] and SourMash [21] can compare genomes more quickly, but are not suited for finding small contaminating sequencing within a larger genome."*

+ There is a potential problem with directionality of contamination. E.g. it is sometimes difficult to know which genome contains the contaminated sequence when there are cross-kingdom matches. The authors' analyses address this question sufficiently and cleverly, and the protein sequence analysis technique addresses this question by predicting kingdoms with fewer members in the cluster as contaminated. However, the other portions of conterminator do not, and if this is not addressed by a similar method as is used by the protein sequence analysis, should be highlighted in the text as a significant shortcoming of the method.

*Abundance based contamination prediction can lead to wrong directionality calls. We added a note to point this out. It reads:*

*“For example, Human repeats cause contamination in multiple bacterial genomes [2]. In this case abundance-based directionality prediction would wrongly call the human genome to be contaminated.”*

*We already explain (p. 7) that for DNA contamination, we use the heuristic that the shorter sequence is usually the contaminating one. We added a sentence with this caveat: "Note that in the rare cases where a contaminating sequence is longer than 20 kb, our method will fail to identify it. However, this prevents us from labeling recent horizontal gene transfer events as contamination. "*

+ There is no discussion of cross-kingdom horizontal gene transfer until the methods section, and what these events may look like to conterminator. It would be helpful to have this discussion moved forward in the paper, and to see what cross-kingdom horizontal gene transfer looks like to conterminator.

*We are very familiar with the literature on horizontal gene transfer, and this topic opens a large can of worms. We intentionally kept our reference to this issue very brief, because we don't want to pursue what would be a lengthy digression, which isn't the main focus of our paper. In general, HGT will not be detected by Conterminator because of the 20 kb length threshold, and because it only looks at isolated contigs as potential contaminants.*

**Second round of review**

**Reviewer 2**

In "Terminating contamination: large-scale search identifies more than 2,000,000 contaminated entries in GenBank", the authors discuss a new method called Conterminator that identifies cross-kingdom contamination in genome assemblies. To date, the majority of published methods used to identify genomics contamination require the user to go back to the reads from the original sequencing data, and thus are computationally intensive and intractable for large-scale analyses (e.g. searching for contamination in all of GenBank). The authors present a tool that addresses this gap, and quickly finds

contamination in genomes and assemblies. This tool is needed in the community, and the analyses presented in the paper shed much needed light on contamination issues in reference databases. The tool is conda installable, and the github repo README is clear enough for a user to begin using the tool. We also find it helpful that lists of contaminants were made available by the authors.

We thank the authors for their revisions, and have no further suggestions.
